# Aneurysms of Pancreaticoduodenal Artery due to Median Arcuate Ligament Syndrome, Treated by Open Surgery and Laparoscopic Surgery

**DOI:** 10.1155/2019/1795653

**Published:** 2019-01-13

**Authors:** Satoshi Tokuda, Shunsuke Sakuraba, Hajime Orita, Mutsumi Sakurada, Tomoyuki Kushida, Hiroshi Maekawa, Koichi Sato

**Affiliations:** Department of Surgery, Juntendo Shizuoka Hospital, Shizuoka, Japan

## Abstract

**Introduction:**

There are many possible causes of an abdominal visceral aneurysm, including the obstruction of the celiac artery by the median arcuate ligament (MAL). We report two cases of an aneurysm of the pancreaticoduodenal artery due to MAL syndrome that we treated surgically.

**Case Presentation:**

Case 1: a 66-year-old Japanese woman was diagnosed with a rupture of an aneurysm of the inferior pancreaticoduodenal artery. Because of the difficulty of endovascular therapy, we performed an emergency operation. We chose an abdominal operation, and the postoperative course was uneventful. Case 2: a 75-year-old Japanese man presented at our hospital with acute abdominal pain, nausea, and cold sweat. Our experience of treating MAL syndrome in case 1 enabled us to diagnose the disease accurately. We chose laparoscopic surgery, and the postoperative course was uneventful.

**Discussion:**

There are several treatment choices for an aneurysm of the pancreaticoduodenal artery due to MAL syndrome. We have performed only a release of the MAL for treatment, but it is difficult to conclude whether only releasing the MAL is enough to ensure a positive long-term prognosis. Regular follow-up is needed in such cases.

**Conclusion:**

Laparoscopic surgery can be considered one of the options for MAL syndrome.

## 1. Introduction

Abdominal visceral aneurysms are rare, accounting for approx. 1% of all aneurysms. It was reported that 60% of abdominal visceral aneurysms occur in a splenic artery, 20% in a hepatic artery, 10% in the superior mesenteric artery, and 2% in the pancreaticoduodenal artery [1]. A ruptured abdominal visceral aneurysm has a poor prognosis; the mortality rates are 25% for a splenic artery, 35% for a hepatic artery, and 50% for a pancreaticoduodenal artery. There are several possible causes of an aneurysm of a pancreaticoduodenal artery: arteriosclerosis, pancreatitis, trauma, fibromuscular dysplasia, segmental arterial mediolysis, and stenosis or obstruction of the celiac artery by the median arcuate ligament (MAL). The MAL is in front of the aortic hiatus, and “MAL syndrome” occurs when the root of the celiac artery is compressed by the MAL. The clinical features of MAL syndrome are chronic postprandial abdominal pain, epigastric bruit, and weight loss [2].

The first reports of MAL syndrome were by Harjola in 1963 and Dunbar in 1965 [3]. MAL syndrome occurs in 2 per 10,000 patients [4] and is more common in young people and thin women. The lower level of the MAL (especially during expiration) or the upper level of the celiac artery (or both) can cause MAL syndrome, which can have nonspecific symptoms, making the diagnosis difficult. A rupture of an aneurysm sometimes reveals MAL syndrome.

Here, we report two cases in which an aneurysm of the pancreaticoduodenal artery due to MAL syndrome was treated successfully by surgery. The first patient underwent a laparotomy, and the other patient underwent laparoscopic surgery. The usefulness of laparoscopic surgery has been reported, including descriptions of more rapid clinical improvement, less complications, quick recovery, and a good cosmetic outcome [5].

## 2. Case 1

Case 1 was a 66-year-old Japanese woman who had presented at another hospital with acute abdominal pain which she experienced when she took a bath before she was rushed to the hospital. Her vital signs indicated shock (systolic blood pressure was 70 mmHg), and her CT scan showed intraperitoneal bleeding. She was transferred to our hospital. She had no notable medical history. On admission, her body temperature was 37.4°C and her pulse was 118 bpm/min.

**Figure 1:**
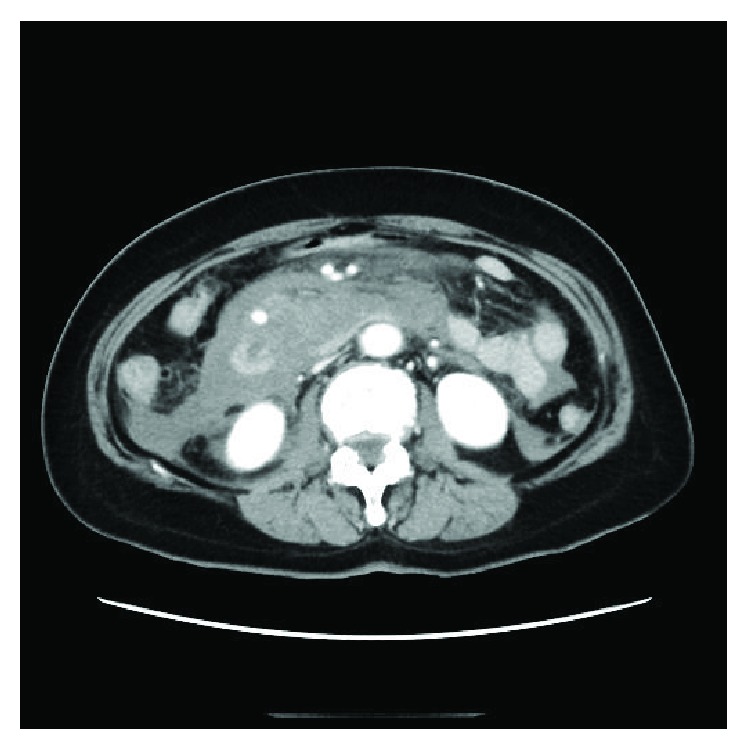
Case 1: a 66-year-old woman, CT scan showing a large amount of ascites and an aneurysm.

**Figure 2:**
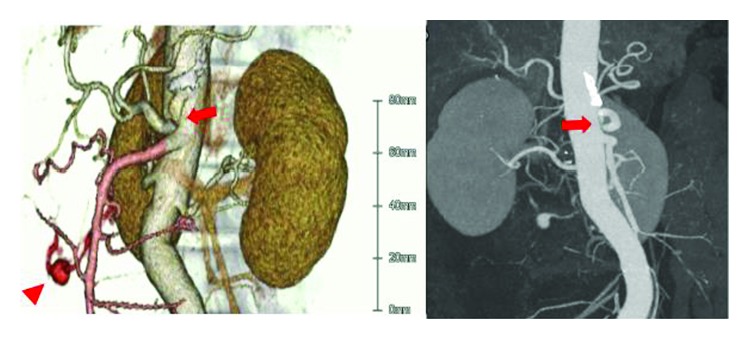
Case 1: the angioarchitectonic revealed the aneurysm of the inferior pancreaticoduodenal artery (IPDA; arrowhead) and stricture of a root of the celiac artery (arrows).

**Figure 3:**
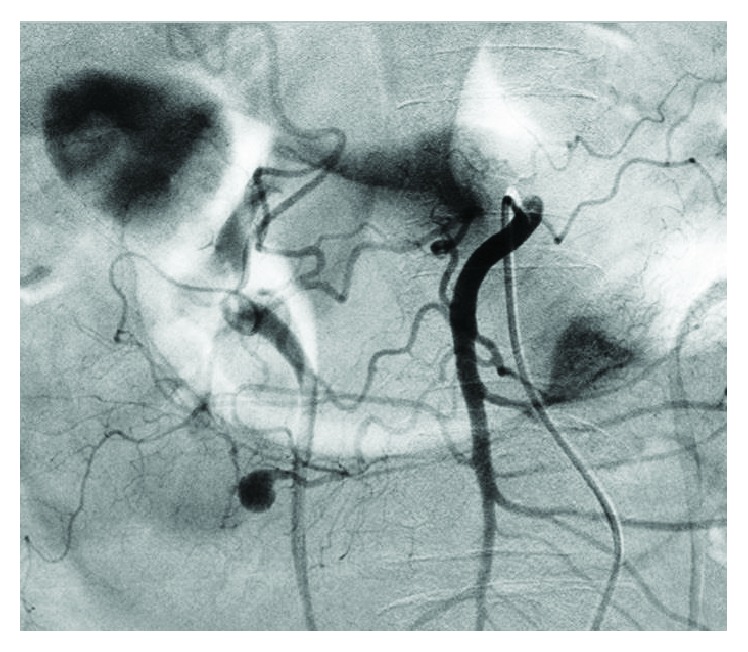
Case 1: angiogram showing an aneurysm of the IPDA.

After fluid resuscitation, the patient's blood pressure was 129/94. Laboratory findings showed slight leukocytosis (15,600/*μ*l) and anemia (9.9 g/dl). A CT scan revealed a great volume of ascites and an aneurysm (Figure 1). The angioarchitectonic examination showed the aneurysm of the inferior pancreaticoduodenal artery (IPDA) and stricture of a root of the celiac artery (Figure 2). We then performed angiography (Figure 3), which also showed the aneurysm of the IPDA. We attempted coil embolization, but because of the difficulty inserting the catheter, we aborted the embolization and decided to perform surgery instead. We had no prior experience with MAL syndrome, and in light of the emergency, we chose an abdominal operation.

We conducted an abdominal median section and could see the intra-abdominal hemorrhage. Excessive bleeding was observed in the retroperitoneum around the duodenum, pancreas, and transverse colon. The IPDA, which is the vascular arcade of the inferior margin of the pancreas, had a 10 mm aneurysm. We confirmed the aneurysm's existence by perioperative sonography, ligated the feeder, and removed the aneurysm.

For the prevention of a rerupture of the aneurysm, we attempted to resect the MAL. After taping the left gastric artery, we observed that the celiac artery was covered by the MAL. We cut the MAL away little by little and confirmed the increase of the beating of the left gastric artery. After placing drainage tubes in the left subphrenic area and the inferior side of pancreas, we closed the wound of the abdominal incision. The operation time was 4 hours 27 minutes, and the blood loss was 1800 ml. The patient began to take hospital meals on postoperative day 8. The postoperative course was uneventful, and she was discharged on the 22nd hospital day. CT scans have shown no recurrence of the aneurysm for 3 years.

## 3. Case 2

Case 2 was a 75-year-old Japanese man who presented at our hospital with acute abdominal pain, nausea, and cold sweat. His CT scan showed retroperitoneal bleeding (around the pancreas and the dorsal side of the ascending colon). His general condition was stable, but he was admitted to our hospital as a conservative measure. His angiography (6 days after admission) showed an aneurysm of the pancreaticoduodenal artery without active bleeding.

**Figure 4:**
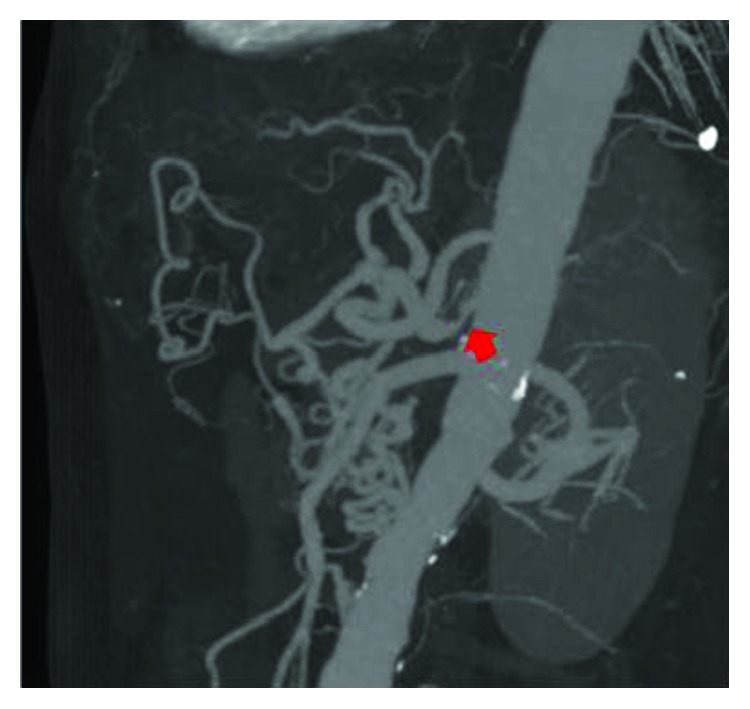
Case 2: the CT scan shows stricture of a root of the celiac artery by MAL (arrow).

**Figure 5:**
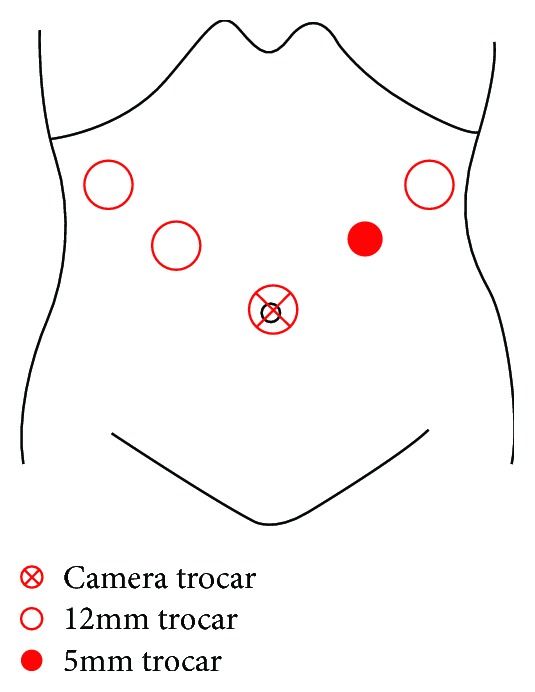
The trocar placement for the laparoscopic surgery for case 2, a 75-year-old man.

Our experience treating MAL syndrome in case 1 enabled us to diagnose the disease accurately in case 2. MAL syndrome was the cause of the aneurysm in this patient too (Figure 4). We selected laparoscopic surgery based on the MAL syndrome and the benefits of this surgery. The patient's posture for the surgery was the lithotomy position. Intra-abdominal pressure of 12 mmHg was maintained. The points of the trocars were as follows: a 12 mm trocar at the navel for the camera, two 12 mm trocars at the right upper abdomen, and a 12 mm trocar and a 5 mm trocar at the left upper abdomen (Figure 5).

First, we lifted the liver umbilical ligament by surgical sutures and put in an organ retractor to the crus of the diaphragm in order to improve the field of vision. After opening the omental bursa, we lifted the stomach with a snake retractor and observed the dorsal side of the stomach. We confirmed the left gastric artery and tied it with tape. The tape was taken out from the right outside trocar, and an assistant pulled it to provide traction of the surgical field. Following the celiac artery to the root, the artery was fastened by the MAL. We cut the MAL away little by little with a vessel-sealing system until the running direction of the celiac artery was clearly confirmed. Using a blood flow meter, we confirmed the improvement of blood flow of the left gastric artery (from 5 mm/min to 69 mm/min). A drainage tube was placed in the left subphrenic area, and the wound of the abdominal incision was closed. The operation time was 3 hours 35 minutes, and the blood loss was minimal at 15 ml. The patient's CT scans have shown no recurrence of the aneurysm for 2 years.

## 4. Discussion

One of the mechanisms of the formation of an aneurysm of the IPDA by MAL syndrome is as follows. (1) The celiac artery is compressed by the MAL, (2) the blood flow to the liver or spleen is decreased, and (3) to compensate for this decrease, the blood flow of the superior mesenteric artery is increased, as is the flow of the IPDA. The IPDA is not very thick, and it cannot bear much blood flow.

However, the precise mechanism underlying MAL syndrome is unclear [6]. The MAL should be considered in the cases of patients for whom a pancreaticoduodenectomy (PD) is planned. In a PD, a compressed celiac artery is the cause of a serious hepatobiliary complication because of the resection of the pancreaticoduodenal arcades. MAL is found in approx. 10% of preoperative CT examinations of patients for whom a PD is planned [7].

There are several choices of treatment for an aneurysm of a pancreaticoduodenal artery due to MAL syndrome: surgery (open, laparoscopic, or robot-assisted [8, 9]), stenting for the celiac trunk, and coil embolization for the aneurysm. In case 1, the difficulty of inserting the catheter for the pancreaticoduodenal artery prevented treatment by an endovascular procedure. Surgeons must therefore be prepared to perform an emergency operation at any time for the treatment of an aneurysm.

The first description of laparoscopic surgery for MAL syndrome was published in 2000 [10]. Since then, several studies have addressed the advantage of laparoscopic management of MAL syndrome: rapid clinical improvement, less complications, quick recovery, decreased rate of postoperative adhesions, and a good cosmetic outcome [4–11]. However, there are negative aspects of laparoscopic surgery. In 9.1% of a laparoscopic group, open conversion was necessary because of bleeding [4]. Depending on the situation, a laparotomy procedure is necessary for the treatment of MAL syndrome.

In our two patients, we performed only a release of the MAL as treatment, and there has been no recurrence of the aneurysms. Other studies described stenting the vessels with operation and angioplasty to prevent recurrence [5, 12, 13]. Because of the relatively short periods of time since the surgery in our patients (2 and 3 years), it is too soon to conclude that only the release of the MAL was enough for a good long-term prognosis. In case 2, we checked the improvement of the blood flow of the left gastric artery. If this improvement had not been confirmed, a reconstruction of the celiac artery might have been necessary. Regular follow-up is needed in similar cases.

## 5. Conclusion

Based on our experience, laparoscopic surgery for MAL syndrome can be considered a good choice. Surgeons should become familiar with the pathophysiology of MAL syndrome and its appropriate treatment.
